# AAPM Report 373: The content, structure, and value of the Professional Doctorate in Medical Physics (DMP)

**DOI:** 10.1002/acm2.13771

**Published:** 2022-09-15

**Authors:** Jay W. Burmeister, Charles W. Coffey, John D. Hazle, Neil Kirby, Yu Kuang, Michael A. Lamba, Brian Loughery, Niko Papanikolaou

**Affiliations:** ^1^ Karmanos Cancer Center/Wayne State University Detroit Michigan USA; ^2^ Vanderbilt University Medical Center Nashville Tennessee USA; ^3^ University of Texas MD Anderson Cancer Center Houston Texas USA; ^4^ University of Texas Health Sciences Center San Antonio San Antonio Texas USA; ^5^ University of Nevada Las Vegas Nevada USA; ^6^ University of Cincinnati Cincinnati Ohio USA; ^7^ William Beaumont Hospital ‐ Dearborn Dearborn Michigan USA; ^8^ University of Texas Health Sciences Center San Antonio San Antonio Texas USA

**Keywords:** DMP, medical physics graduate education, professional doctorate degree

## Abstract

The Professional Doctorate in Medical Physics (DMP) was originally conceived as a solution to the shortage of medical physics residency training positions. While this shortage has now been largely satisfied through conventional residency training positions, the DMP has expanded to multiple institutions and grown into an educational pathway that provides specialized clinical training and extends well beyond the creation of additional training spots. As such, it is important to reevaluate the purpose and the value of the DMP. Additionally, it is important to outline the defining characteristics of the DMP to assure that all existing and future programs provide this anticipated value. Since the formation and subsequent accreditation of the first DMP program in 2009–2010, four additional programs have been created and accredited. However, no guidelines have yet been recommended by the American Association of Physicists in Medicine. CAMPEP accreditation of these programs has thus far been based only on the respective graduate and residency program standards. This allows the development and operation of DMP programs which contain only the requisite Master of Science (MS) coursework and a 2‐year clinical training program. Since the MS plus 2‐year residency pathway already exists, this form of DMP does not provide added value, and one may question why this existing pathway should be considered a doctorate. Not only do we, as a profession, need to outline the defining characteristics of the DMP, we need to carefully evaluate the potential advantages and disadvantages of this pathway within our education and training infrastructure. The aims of this report from the Working Group on the Professional Doctorate Degree for Medical Physicists (WGPDMP) are to (1) describe the current state of the DMP within the profession, (2) make recommendations on the structure and content of the DMP for existing and new DMP programs, and (3) evaluate the value of the DMP to the profession of medical physics.

## 1 BACKGROUND

In 2014, completion of an accredited residency training program became a requirement for eligibility for the American Board of Radiology physics examination. The announcement of this change in 2007 threatened to significantly reduce the number of board‐eligible trainees since there were fewer than 20 accredited residency programs at that time.^1^ The Professional Doctorate in Medical Physics (DMP) was created as a solution to the shortage of structured training opportunities necessary to fulfill clinical needs in medical physics, and the first program began at Vanderbilt University in 2009. This program was subsequently accredited by CAMPEP in 2010. Since then, four more programs have become accredited, including the University of Cincinnati, the University of Texas Health Science Center San Antonio, the University of Nevada Las Vegas, and Wayne State University.

The American Association of Physicists in Medicine (AAPM) established the Working Group on the Development of a Professional Doctorate Degree (WGPDMP) in 2008 to evaluate the impact of this degree and keep membership updated on developments thereof.^2^ This group produced a white paper on the DMP entitled “The Impact of the Professional Doctorate (PD) in Medical Physics.” This document evaluated many facets of the creation of the DMP including impacts on the profession, on existing academic programs, on medical physics research, and on those already board certified and practicing with a Master of Science (MS) degree. Since that time, over 70 students have completed the DMP. More importantly, the number of conventional residency positions has grown substantially. For example, from 2009 to 2016, the number of CAMPEP‐accredited residency positions available each year increased from 60 to 144.^1^ Nearly 90% of these positions were in radiation therapy physics, and that number began to quickly approach the estimated demand for radiation therapy physicists in 2020.^3^ Fewer than 10 of these positions were within DMP programs. That trajectory has continued and one may argue that we now have enough conventional residency positions to meet clinical demand without existing DMP positions. However, a recent evaluation of supply and demand for radiation oncology physicists suggests that this number may have to grow substantially to meet predicted demand in 2030.^4^ We do not currently have published estimates of clinical demand for diagnostic imaging physicists.

Since the DMP was originally conceived to provide a solution to the shortage of residency training positions, and this shortage appears to have largely been satisfied through conventional residency training positions, it is now important to reevaluate the purpose and the value of the DMP. Moreover, it is important to outline the defining characteristics of the DMP to assure that all programs provide this anticipated value. The relatively slow growth of DMP programs since their inception also suggests that there may be a need for reevaluation, evolution, and/or redefinition of the degree pathway. As such, the WGPDMP initiated this report to (1) describe the current state of the DMP in the profession, (2) make recommendations on the structure and content of the DMP for existing and new DMP programs, and (3) evaluate the value of the DMP to the profession of medical physics. This report does not address the question of whether the DMP should be a part of our future educational infrastructure. It is our hope that i will initiate additional discussion and ultimately action by our profession regarding both the characteristics of the DMP and its value to our profession.

### 1.1 Current state of the DMP

To date, the AAPM has not created guidelines or recommendations for the content or characteristics of the DMP. As a result, there is significant heterogeneity across existing programs. Accreditation of these programs is based on CAMPEP standards, and while these are established independently, CAMPEP does consider AAPM recommendations in the creation of these standards and uses AAPM‐developed content guidelines in this process. Current standards include curricular recommendations from AAPM Report 197 for the didactic component of the DMP and those of AAPM Report 249 for clinical training.^6^, ^7^ While fulfillment of the recommendations of AAPM Reports 197 and 249 represents a minimum standard, most programs consist of significantly more than this minimum standard. For example, four of five accredited DMP programs provide training beyond the recommended core elements of an MS degree and clinical residency training as described by AAPM Reports 197 and 249, respectively.

To provide the data necessary for recommendations for standardization and for determination of the potential impact of the DMP on trainee output, two surveys conducted by the WGPDMP are presented here. These surveys gathered data from program directors at universities with existing or proposed DMP programs as identified by self‐response on the annual CAMPEP graduate program survey. The first survey was conducted in September 2016, and data supplied by eight institutions were presented at the 2017 AAPM Annual Meeting.^5^ The second was conducted in September 2020, and responses were collected from six programs (in the intervening years, three programs decided not to continue pursuing the DMP, and one new program expressed interest in pursuing the DMP). Both surveys included data from all five accredited DMP programs. The initial survey gathered data on the breadth and scope of the existing or proposed program, student statistics (if any), and aspects of tuition and funding. The second survey gathered these data along with information on program length, didactic training requirements and offerings, research training, and matriculation and graduation data. Table 1 provides selected data from the 2020 survey for the five currently operational programs. Data from the sixth respondent are not included since the program is not yet in a state mature enough to provide responses to most survey questions. Complete results from both surveys are provided in the Appendix.

**TABLE 1:** Selected data from the five accredited programs provided within the 2020 WGPDMP survey

	1	2	3	4	5	Ave
Total credit hours required for the DMP	90	80	92	118	98	95.6
Credit hours representing the 2 years of clinical training	30	40	36	44	52	40.4
Required research credit hours	12	4	6	17	1	8.0
Credit hours of didactic coursework	48	40	50	57	45	48.0
Credit hours required for MS degree	35	40	44	58	NA	44.3
Credit hours required for PhD degree	90	NA	NA	NA	72	81.0
Professional skills training beyond that provided in MS/PhD	Yes	No	No	Yes	Yes	
Minimum time to complete the DMP (y)	4	3.75	4	4	4	4.0
Average time to complete the DMP (y)	6	3.75	4	4	4	4.4
Average number of students matriculating per year	0	2	1	3	3	1.8
Maximum number of students that could matriculate per year	3	3	1	6	4	3.4
Total tuition for entire program for in‐state student	$70k	$40k	$150k	$116k	$84k	$92k
Total tuition for entire program for out‐of‐state student	$125k	$65k	$150k	$210k	$144k	$139
Total graduates as of 9/2020	1	4	42	10	13	

Abbreviations: DMP, Professional Doctorate in Medical Physics; NA, not applicable.

While the number of CAMPEP‐accredited programs increased from three to five between the two surveys, there were no substantial differences in total credit hours or in didactic, research, or clinical training observed between the two surveys. The number of total graduates increased from 29 to 70 between the two survey dates; however, the number of DMP students enrolled decreased from 42 to 25, and the number of annually matriculating students decreased from 11 to 9.

Existing DMP programs require an average of 95.6 credit hours for the entire degree. This total credit hour requirement is similar in breadth to typical Doctor of Philosophy (PhD) programs and, as shown in Table 1, is greater than the average number of credit hours required for the PhD at these institutions. If one assumes that the MS degree contains only the didactic requirements and research recommendations from CAMPEP and from AAPM Report 197, the number of credit hours for the MS degree may be considered to represent the current minimum educational requirements. The average number of credit hours for the MS degree at these institutions is 44.3, which is approximately 25% less than the average number of didactic and research credit hours for the respective DMP programs of 56 credit hours. However, since the DMP program at one of the four institutions with an MS degree does not provide training beyond the MS plus residency, this average masks the substantial differences at the other programs. In fact, the total didactic and research credits for one of the DMP programs is more than 70% greater than for its respective MS degree. For the three programs that have an MS degree and assert that they contain more than the minimum educational requirements, the average number of didactic and research credit hours for the DMP is 40% higher than the credit hours required for their respective MS programs. While one can conclude that, in general, the required workload is higher for the DMP student during the didactic/research component of the program than an MS student at the same institution, some institutions also require coursework during additional terms (for example, spring or summer terms) during which MS students may not take courses. DMP programs must assure that additional coursework required for DMP students does not diminish the breadth of the clinical training component of the program. For simplicity, we provide and discuss average credit hour values as shown in Table 1. Since different institutions have different academic calendars, the meaning of a credit hour differs across institutions. Quantitative comparison of absolute credit hours should technically only be performed within each institution; however, the trends observed within each individual institution are very similar to the average trends presented and discussed above. While most existing DMP programs offer additional content beyond the minimal training requirements, the nature of this content should confer an advantage to the DMP graduate for this degree to add value to our profession.

### 1.2 DMP structure and content

For the DMP to remain an important training pathway for clinical medical physics, it must arguably add value that is not currently achievable through the traditional training programs (MS + residency or PhD + residency). Since it is a doctorate degree, it must have breadth, depth, and scope similar to other doctoral degrees. As such, we believe that the DMP should be approximately commensurate in breadth with the PhD, our other doctoral degree. However, it is in some sense more closely related to the MD degree in that it provides specialized clinical training rather than research training. Improvements in patient care and treatment outcome can result from both the development of new medical techniques, as might be anticipated from the research training we provide to our PhD trainees, but also from refinements of existing processes, as we might anticipate from the clinically oriented research and development training provided to DMP trainees. The completion of didactic coursework satisfying AAPM Report 197 and clinical training satisfying AAPM Report 249 should not be considered sufficient for a DMP since it is neither commensurate with the breadth of a PhD nor does it provide unique training that does not already exist within our current training infrastructure. As such, the DMP should contain coursework credits commensurate with existing doctorate programs, thus providing the trainee with substantially more than the education and training of an MS degree plus 2‐year clinical training program. It should be noted that most existing DMP programs do provide training significantly beyond an MS plus 2‐year of clinical training, as shown in Table 1.^5^ Specific examples of this training identified by individual programs currently include additional research experiences, seminar courses in leadership and quality improvement, and additional didactic coursework in physics, mathematics, biomedical engineering, computer science, biology, business, and education. The complexity of medical physics practice continues to increase, while simultaneously, initiatives such as MedPhys 3.0 endeavor to expand the applications of physics to medicine. It is difficult for graduate programs to add new coursework to meet expanding needs while continuing to provide existing coursework. The DMP provides an infrastructure within which to expand the opportunities for this enhanced training. While this additional coursework will advance the capabilities of DMP graduates, it may be most valuable to develop specific areas of focus to more clearly distinguish this pathway. For example, a specific focus on hospital administration, along with explicit recognition of this distinction on the degree itself, could more clearly define the value of the degree for those outside of the profession of medical physics.

Finally, it is important to recognize that the DMP, like other professional degrees, provides a different learning environment and sets different goals for the student. It also appeals in principle to a different student population. Of course, candidates desiring a career in research will continue to pursue the PhD pathway. However, some medical physics trainees currently undertake a PhD in part to assure a higher probability of obtaining such clinical training in the form of an accredited residency. In contrast, the emphasis throughout the DMP program is on preparation of clinical scientists rather than researchers. This should include training in clinical protocol development, quality improvement, risk analysis, etc., and clearly differentiates the DMP path from the PhD path. The DMP candidate seeks a career in clinical medical physics but would also benefit from the additional training in teaching methods, clinical mentoring, and clinical research to support appointment as clinical faculty. Additionally, if we are to rethink the training of our clinical workforce, we may decide that a structure which integrates didactic and clinical training throughout the program is advantageous over sequential delivery. One DMP program has already implemented this integrated structure and provides clinical experience beginning in the first year of the program, offered in conjunction with didactic education. While the experiential learning involved in performing independent research and managing an in‐depth research project helps many PhD physicists develop tools that help them become leaders in our field, the additional leadership training and clinical and research projects that should be included in the DMP could provide similar advantages. It may be beneficial to require that DMP candidates obtain focused training emphases, such as the equivalent of a minor in a particular subject (e.g., computer science, information science, hospital administration). Additionally, if specific skills are identified as an expected advantage of the DMP, such as leadership capabilities, then a specific list of content areas should be stipulated.

The DMP was initially proposed as a mechanism to create additional residency positions beyond existing hospital‐funded positions. The funding burden for these new positions was transferred from the hospital to the student. Thus, a significant aspect of concern with the DMP is the overall cost associated with the degree. The mean cost for the entire DMP degree is approximately $92,000 (range $40,000–$150,000) for students paying in‐state tuition and approximately $139,000 (range $65,000–$210,000) for students paying out‐of‐state tuition. Financial concerns include the potential discouragement of high‐quality candidates from entering the field due to the prohibitive cost and the concern that future entrants might be disproportionately biased toward those who can afford the cost of the DMP. Currently, underrepresented minorities make up 8% of all DMP graduates and students, which is similar to the current estimated AAPM membership level of 6.5%.^8^ Women currently make up 26% of DMP graduates and students, which is similar to the current AAPM membership level of 23%.^8^ However, we should continue to monitor these data to assure that they are not negatively affected by the DMP. An additional important financial concern of the DMP is the possibility that it could threaten our conventional residency financial support by facilitating a student‐funded training model. Indeed, some institutions with a DMP program also have conventional residency positions in which residents are paid to do the same work for which DMP students are paying tuition. It is critical for our profession to retain these established training positions. In addition, we will have a greater opportunity to attract the best and brightest if we minimize their training cost, and it is advantageous to keep our parallels with the physician training pathway in terms of financial support for residency training.

A resulting question that must be answered is whether the student‐funded clinical training pathway must continue to exist. While this financial burden is similar in nature to the burden our physician colleagues carry when pursuing their professional doctorate, the potential consequences of this financial burden must be carefully evaluated. If we, as a profession, find these consequences too problematic, we must find a way to create funding for the clinical training component for all DMP positions. One mechanism to achieve this is to financially reimburse the DMP students for their clinical service during the program. Several institutions have established such a mechanism, providing funding to the student either during their clinical years or distributing it across the entire degree program. In this manner, the financial burden of the DMP may be significantly offset.

If the DMP is to become a permanent component of the medical physics education and training infrastructure, not only is it necessary to define its recommended composition and characteristics more clearly but also to demonstrate its value beyond, and distinct from, our existing pathways. Establishing such standards would help avoid confusion outside of our profession about the meaning of the DMP and more firmly establish its value within the profession.

### 1.3 Value of the DMP

With the rapid growth in technical complexity in the medical specialties supported by medical physics as well as the increasing roles in which medical physicists are often required to serve, additional graduate training could provide significant value. There are many skills that the clinical physicist could obtain which would ultimately benefit the institutions that employ them and the patients they serve. While an important question that was asked in the early days of DMP development was, “What are the minimum training requirements for a DMP?” a more relevant question now may be, “What is the best way to train a DMP student to meet the evolving needs of the clinical medical physics profession for 2020 and beyond?” The DMP might serve our profession as a mechanism to introduce an integrated medical physics training experience that best prepares the graduates for clinical employment.

The DMP may offer several valuable characteristics that can both enhance the training experience of entrants into the medical physics profession and produce more valuable and better prepared trainees. These include the possibility of a holistic learning environment in which students experience topics in the clinic at the same time they are covered in the didactic coursework as well as the integration of professional skills development such as leadership, ethics, project management, business and computer skills, and the opportunity to interact and communicate with patients, other medical professionals, hospital leadership, and vendor specialists. While a typical PhD plan of work includes elective coursework that enhances the student's research capabilities and perspective, the DMP additionally offers the possibility of incorporating a variety of useful electives and tools that may not be considered relevant to a PhD student's research. Some examples of these include scientific coursework beyond the scope of relevance of the student's PhD research, including physics, mathematics, biology, computer science, and engineering, for example. Additional examples include training in education, business, and clinical communication. Incorporation of cognitive science and modern teaching methodologies is an important initiative in medical physics education. The medical physicist provides an important contribution to the business and financial operations of the hospital. Clinical communication skills are critical as many medical physicists move into more patient‐facing roles in clinical practice. Medical physicists commonly participate professionally in these areas and historically have done so without formal training. The DMP can facilitate a more comprehensive education and training program that can potentially produce a more well‐rounded and valuable medical professional.

For students who wish to pursue a career in research, the PhD remains the required pathway; thus, we would not expect the DMP to draw students from the PhD pathway. Anecdotally, the PhD pathway has also sometimes served as a mechanism for career advancement since many leadership positions require a terminal doctorate. It may be that the DMP could fill this niche in the future, as it will arguably provide better preparation for such leadership and management roles than intensive research training. Explicit recognition of this focus, as suggested previously, would help promote this purpose. In addition, this could potentially help preserve our mentoring resources for those who wish to do research in our field. Our PhD physicists are a very valuable asset to our clinical infrastructure, and they will still have the conventional residency training pathway for their clinical training.

As an associated point, the DMP has traditionally contained a guaranteed clinical training position within the program. This served the initial purpose of the DMP and made it attractive to graduate education candidates despite the high cost. However, one may question whether the guarantee of the accredited clinical training component is still necessary as a core element of the DMP degree. A method by which the student competed for the accredited clinical training component would help eliminate any negative perception of the degree associated with whether the DMP student acquired their clinical training spot by merit or by financial capability. Lastly, it would help further promote the success of the MedPhys Match process. Conversely, in an integrated training model, the clinical training must necessarily be guaranteed, as it is an integral part of the entire program. Furthermore, this structure may be more appealing to medical physics candidates since it does not carry the possibility that they successfully complete a graduate degree but are not subsequently able to secure accredited clinical training and thus board eligibility. One important associated consideration is therefore the selection process for entrants into a DMP program. While traditional medical physics graduate student selection criteria still apply, additional emphasis must be placed on topics such as empathy, self‐awareness, emotional intelligence, integrative thinking, prior clinical and/or research experience, and potential for clinical excellence. However, assessment of these attributes is more challenging before graduate study than afterward.

These possibilities and provisions, along with others which may potentially be identified later, should help build uniformity and standardization of the definition of the DMP degree and open possibilities for additional programs. The 2020 WGPDMP survey results presented in Table 1 show a total of 70 graduates from accredited DMP programs. Of these, 67 held clinical appointments, and three held academic appointments at the time of the survey. While some DMP graduates currently serve in faculty positions, not all academic institutions currently recognize the DMP as fulfilling the requirements for a particular faculty position. Building a clearer understanding of the meaning and significance of the DMP may help overcome these current limitations and help establish its value within the profession. For example, an emphasis on leadership and hospital administration may establish the DMP as an alternative to an MBA for such leadership positions. Finally, a comparison of American Board of Radiology (ABR) pass rates for DMP students and graduates is an instructive evaluation of both the quality of DMP students and their respective training. ABR initial certification examination first‐time pass rates for DMP graduates for parts 1, 2, and 3 are currently 82%, 91%, and 71%, averaged over all institutions. These compare favorably with pass rates for all examinees, which are 62%, 87%, and 64% averaged over 2016–2019^9^ (The 2018 ABR part 1 exam data were excluded due to an abnormality in the exam.). However, this may be related more to these particular institutions than to the DMP itself. Average part 1 pass rates for non‐DMP graduate students and parts 2 and 3 pass rates from residency graduates from these same institutions over the same period are 75%, 97%, and 80%. Since the relative fraction of non‐DMP examinees from each institution is different from that of DMP examinees, these average DMP and non‐DMP pass rates cannot be directly compared. In addition, these results are based on a relatively small cohort at a small number of facilities, and it is therefore difficult to draw any clear conclusions.

## 2 SUMMARY AND RECOMMENDATIONS

This report describes the potential value of the DMP to our educational infrastructure and to our profession and the corresponding recommendations for the content and structure of the DMP to best realize this potential. If the primary purpose of the DMP is to elevate the professional preparation, usefulness, and status of our trainees, we should ensure that the DMP adds value to our existing education and training infrastructure. In addition, neither student‐funded training positions nor guaranteed clinical training positions are necessary to serve these aims and should be considered carefully to determine how they affect our educational infrastructure. The primary recommendations of this white paper are summarized below.

### 2.1 Content of the DMP

To differentiate the DMP from the existing MS + residency training pathway and to assure that it adds value to the profession and to medicine, the DMP must contain more than typical MS‐level didactic training and 2‐year of clinical training. The scope and extent of the DMP should be commensurate with other doctoral degree programs, including the PhD. Additional coursework included in the DMP should be designed specifically to provide the greatest value to the practicing clinical medical physicist and should include both didactic components and clinical research. Specific content areas identified as conferring anticipated advantages to the DMP graduate should be identified and included in DMP program curricula. In addition, it may be beneficial to require that DMP candidates obtain focused areas of training, such as the equivalent of a minor in a particular subject (e.g., computer science, information science, finance). Didactic medical physics curricula currently focus primarily on the physics, biology, and technology relevant to the field. These topics are essential, but they do not cover the full breadth of professional skills needed to practice medical physics. Other skills such as leadership, quality improvement, healthcare operations, and project management are critical to maximize our contribution to medicine. The DMP can add to the quality and value of a medical physics trainee, and there is currently a need to strengthen and standardize this content. DMP programs must assure that additional coursework required for DMP students does not diminish the breadth of the clinical training component of the program.

### 2.2 Funding for the DMP

Clinical training within DMP programs should be institutionally funded to the greatest extent possible. The DMP is a professional doctorate degree. These types of degrees are typically student funded. The primary reason for this is that they are costly programs to run. They require a large amount of one‐on‐one training from highly trained and expensive personnel. Although a free professional doctorate degree may not be a realistic expectation, the continued exploration of these funding scenarios is important to offset DMP student debt and increase accessibility to high quality applicants of all backgrounds. Admissions and graduate data should be monitored to ensure that increasing the financial burden on our applicant pool does not have a negative effect on our education and training pipeline and to determine whether the DMP could potentially exacerbate the existing dearth of underrepresented minority students.

### 2.3 Value of the DMP

The members of our profession should carefully evaluate the merits of the DMP and determine whether the value and professional status of our future trainees is substantially enhanced by the inclusion of this degree pathway. We strongly encourage the leadership and membership of the AAPM to consider, discuss, and make specific recommendations on this issue for the benefit of our future trainees and professional members.

We recognize that many more issues will arise regarding the implementation of the DMP but hope that this report initiates intentional discussion within our field about the need for and potential value of this degree and the current issues facing our educational infrastructure and our trainees. Without such discussion and subsequent efforts to adapt our education and training infrastructure to meet what we believe is in the best interest of our profession and the patients we serve, the DMP will continue to progress without overarching guidance. The DMP was originally created out of necessity, but we need to now re‐evaluate our current situation and carefully discuss its role and its value. Does the DMP have a place in our future education and training infrastructure? If so, what role should it play, and why is this role necessary? As professional medical physicists, we are all stakeholders, and we strongly encourage the membership of the AAPM to participate in these discussions and provide feedback which will help shape the future of our education and training pathway.

## CONFLICT OF INTEREST

The authors declare no conflict of interest.

## AUTHOR CONTRIBUTIONS

All authors substantially contributed to the conception or design of the work; or the acquisition, analysis, or interpretation of data for the work; drafted the work or revised it critically for important intellectual content; gave the final approval of the version to be published; and agreed to be accountable for all aspects of the work in ensuring that questions related to the accuracy or integrity of any part of the work are appropriately investigated and resolved.

## Supporting information



Supporting Information
