# Correction: “AAPM WGPDMP Report 373: The content, structure, and value of the Professional Doctorate in Medical Physics (DMP).”

**DOI:** 10.1002/acm2.14067

**Published:** 2023-06-21

Burmeister JW, Coffey CW, Hazle JD, et al. AAPM WGPDMP Report 373: The content, structure, and value of the Professional Doctorate in Medical Physics (DMP). *J Appl Clin Med Phys*. 2022;23:e13771.

The text of the article title, which originally published online September 15, 2022, Volume 23, Issue 10, as “AAPM Report 373: The content, structure, and value of the Professional Doctorate in Medical Physics (DMP)” was corrected to include the Working Group on the Professional Doctorate Degree for Medical Physicists (WGPDMP) so that the article title has been revised to “AAPM WGPDMP Report 373: The content, structure, and value of the Professional Doctorate in Medical Physics (DMP).” The online version of this article has been corrected accordingly.

