# Supplementary material for: AAPM Report 373: The content, structure, and value of the Professional Doctorate in Medical Physics (DMP)
Source: J Appl Clin Med Phys. 2022 Sep 15;23(10):e13771. doi: 10.1002/acm2.13771 (PMC9588257; doi:10.1002/acm2.13771)
Supplement: Supplementary file 1 — Supporting Information [file ACM2-23-e13771-s001.docx]

**Appendix**

**Complete Survey Data**

**9/2016 Survey Data:** (Listed responses are in the same order as the “Institution Name” response)

**Institution Name (8 responses)**

Wayne State University

Vanderbilt University Medical Center

UT M. D. Anderson Cancer Center

University of Nevada, Las Vegas

Duke University

University of Texas Health Science Center San Antonio

University of Cincinnati

San Diego State University

**Is your DMP program already approved by your institution? (8 responses)**

Yes: 5 (62.5%)

No: 3 (37.5%)

**Is your DMP program accredited by CAMPEP? (8 responses)**

Accredited: 3 (37.5%)

Under review: 1 (12.5%)

Preparing self-study for submission: 1 (12.5%)

Not there yet: 3 (37.5%)

**How many credit hours are required for your DMP (1 credit hour = 1 contact hour per week)? (8 responses)**

90

92 credit hours

96

80 credits

40–45 didactic credits + additional training (proposed)

100

123–127

Planning stage at this time

**How many of these credit hours are required to be research? (8 responses)**

12

6 credit hours

8 hours for a capstone project

4 credits

0–6 (proposed)

0

17

Planning stage at this time

**How many of these credit hours represent the required 2 y of clinical training? (8 responses)**

30

30 credit hours in the last 2 y

48

40 credits

Undecided

50

8

Planning stage at this time

**How many of these credit hours are didactic coursework (excluding research and clinical training)? (8 responses)**

48

48

50 credit hours + 6 credit hours of practicum in the second year

40 credits

35–40 (proposed)

50

63 hours during first 2 y, then ~8 h/wk in classroom setting for years 3 and 4

30 units (10 courses at 3 units each)

**What is the total duration of your DMP program? (8 responses**)

4–5 y

4 y

4 y

4 y (12 academic terms)

4 y (proposed)

4 y

4 y, first summer off

Planning stage at this time

**If you offer an MS degree, how many credits are required to complete the MS degree? (8 responses)**

35

Nonthesis option: 32 didactic credit hours + 6 h of practicum. Thesis option: 26 didactic credit hours + thesis

The master’s phase of our DMP will be 48 semester credit hours including the capstone project

40 credits

41

NA

63

30 units (10 courses at 3 units each)

**If you offer a PhD degree, how many credits are required to complete the PhD degree? (7 responses)**

90

We do not offer a PhD in medical physics

Technically our MS and PhD programs are offered by a different institution. They require a minimum of 42 h for our professional master’s degree and a minimum of 82 h for the PhD

NA

41

96

NA

**Do you consider your DMP program to be more comprehensive than the recommended core elements of an MS degree and clinical residency (as described by AAPM reports 197 and 249, respectively)? (8 responses)**

Yes: 4 (50%)

No: 4 (50%)

**Please identify all specific didactic elective coursework AVAILABLE within your DMP program (check all that apply): (7 responses)**

Physics: 5 (71.4%)

Mathematics: 5 (71.4%)

Computer science: 5 (71.4%)

Biology: 5 (71.4%)

Business: 3 (42.9%)

Education: 2 (28.6%)

Other: 4 (57.1%)

**Please identify “other” if checked: (5 responses)**

Biomedical engineering

Biomedical engineering

In theory, our students can take electives in these areas at neighboring institutions such as Rice University and the University of Houston, and occasionally some do. Most recently, these have been a cancer biology course and a computer science course.

No electives in DMP program

Planning stage only

**Please identify all specific didactic elective coursework REQUIRED within your DMP program (check all that apply): (5 responses)**

Physics: 1 (20%)

Mathematics: 0 (0%)

Computer science: 0 (0%)

Biology: 1 (20%)

Business: 0 (0%)

Education: 0 (0%)

Other: 4 (80%)

**Please identify “other” if checked: (4 responses)**

Sectional anatomy

Core medical physics curriculum

No electives in DMP program

Planning stage only

**How many students have graduated from your DMP program? (8 responses)**

0

0

0

28 (24 in therapy track and 4 in diagnostic track)

NA

One

No DMP graduates yet

We don’t have a DMP program as yet. We have a Master’s in Medical Physics which graduates an average of 6 students/year (for the last 8 y).

**How many students are currently enrolled in your DMP program? (8 responses)**

0

0

15 (11 in therapy track and 4 in diagnostic track)

3

NA

12

12

NA

**How many students currently matriculate each year into your DMP program? (8 responses)**

0

0

3 (2 in therapy track and 1 in diagnostic track)

1

NA

3–5

2–4, with the aim of stabilizing at 4/y

NA

**What is the maximum number of students who could ultimately matriculate each year into your DMP program? (8 responses)**

3

3

2

5 (4 in therapy track and 1 in diagnostic track)

Undecided

5

4

2 with possible extension to 4

**What is the total tuition burden FOR THE ENTIRE DMP PROGRAM for an in-state student? (8 responses)**

66,150

Private institution (no in-state/out-of-state student category): $132,036 (4 y)

We anticipate that it will be around $100,000–$115,000

0

Undecided

72,000

$58,000 tuition

Tentatively we anticipate $50,000

**What is the total tuition burden FOR THE ENTIRE DMP PROGRAM for an out-of-state student? (8 responses)**

124,920

Private institution (no in-state/out-of-state student category): $132,036 (4 y)

We anticipate around $140,000–$155,000

0

Undecided

140,000

$70,000 tuition

Tentatively we anticipate $50,000

**Do you provide funding (scholarships, clinical employment, etc.) for your students during their clinical training? (8 responses)**

Yes

Yes

No

No

Hoping to but not approved yet

Undecided

~$40,000 tuition remission is split among all students. Some occasional clinical support, but nothing promised.

We can provide TA positions in year 1 and 2 of the DMP program

**If yes, please specify the mechanism and amount: (5 responses)**

Scholarship (proposed $10k for resident and $50k for nonresident total for years 3 and 4)

Scholarship (tuition remission of 60%): $18,086.40 per year (Y3 and Y4). Clinical stipend of $10,000 per year (Y3 and Y4).

Graduate Assistantships—$18,000/y

Typically split evenly, but altering this provide less funding years 1/2 and more years 3/4.

An amount of $169,000/year for a 0.5 TA appointment

**Do you have a specific mechanism for a prior MS or PhD graduate from your institution to complete the DMP? (8 responses)**

Yes: 2 (25%)

No: 6 (75%)

**If yes, please describe: (3 responses)**

Currently, graduates must complete all remaining coursework and clinical rotations. We intend to address this later.

To complete the additional coursework (~18 didactic credit hours and 30 clinical credit hours) and research project (6 credit hours).

1 y of didactic coursework (20 credits) + 2 y clinical residency (40 credits)

**Do you allow your DMP students to enter the MedPhys Match? (5 responses)**

Yes: 3 (60%)

No: 2 (40%)

**If not, please explain why: (5 responses)**

Our DMP program includes the 2 y clinical training in our own institution or in the last year at Mayo-Clinic at Jacksonville for one student for the whole year or at the Proton Center at Knoxville for 4 mo. We do not allow other locations for our clinical training currently.

We have not crossed that bridge, yet. Personally, I see no reason not to allow this.

Undecided

DMP students will complete the 2 y residency requirement at UTHSCSA in the third and fourth year of the program.

The details of the DMP has yet to be worked out. We are in the process of being accredited for a residency training program. If that is successful, we will integrate the MS and Residency training program into a new DMP program while maintaining the MS program.

**9/2020 Survey Data:** (Listed responses are in the same order as the “Institution Name” response)

**Institution Name (6 responses)**

Wayne State University

University of Nevada, Las Vegas

Vanderbilt University Medical Center

University of Cincinnati

University of Texas Health Science Center San Antonio

Florida Atlantic University

**Is your DMP program already approved by your institution? (6 responses)**

Yes: 5 (83%)

No: 1 (17%)

**In which specialization(s) do you currently offer a DMP? (5 responses)**

Therapy: 3 (60%)

Imaging: 1 (20%)

Both: 1 (20%)

**Is your DMP program accredited by CAMPEP? (5 responses)**

Yes: 5 (100%)

No: 0 (0%)

**Do you provide training beyond the recommended core elements of an MS degree and clinical residency (as described by AAPM reports #197 and #249, respectively)? (5 responses)**

Yes: 4 (80%)

No: 1 (20%)

**How many credit hours are required for your DMP? (5 responses)**

90

80

92

118

98

**How many of these credit hours are required to be research? (5 responses)**

12

4

6

17

1

**How many of these credit hours represent the required 2 years of clinical training? (5 responses)**

30

40

36

44

52

**How many of these credit hours are didactic coursework (excluding research and clinical training)? (5 responses)**

48

40

50

16

45

**If you offer an MS degree, how many credits are required to complete the MS degree? (5 responses)**

35

40

44

58

N/A

**If you offer a PhD degree, how many credits are required to complete the PhD degree? (5 responses)**

90

NA

NA

NA

72

**Please identify disciplines in which didactic elective coursework is AVAILABLE within your DMP program (e.g., physics, mathematics, computer science, etc.): (5 responses)**

Physics, mathematics, computer science, BME, biology, business, education

Engineering, physics, computer science

Biomedical engineering

Molecular genetics, biochem and microbiology—molecular medicine. Physiology—statistics and experimental design. Mechanical engineering-radiation forensics—radiation detection and measurement. Physics—remedial coursework as required.

Our DMP degree is a fixed curriculum. There are no elective courses.

**Please identify all SPECIFIC didactic elective courses REQUIRED within your DMP program: (5 responses)**

None required

None

NA

Translational molecular medicine in Hem Onc, statistics and experimental design, radiation detection and measurements.

NA

**Do you provide professional skills (e.g., leadership, management, quality improvement) in your DMP that are more comprehensive than for MS or PhD programs (if these exist)? If yes, please describe. (5 responses)**

Yes, additional 2 credit course on professional aspects of MP

No

No, we have a 3 credit ethics/leadership core course recently created

Not formal coursework, but third/fourth year students meet weekly for 1.5 h with faculty during which these issues are often addressed.

We have an annual “Leadership and Vision” seminar series (12 lectures) for our DMP students. They are additionally required to complete an ABR-style quality improvement project to graduate.

**To date, how many students have graduated from your DMP program? (5 responses)**

1

4

42

10

13

**Please provide the professional appointment status of these graduates. [Please provide the number of graduates in each of the following categories: (1) academic appointment; (2) clinical appointment; (3) joint academic/clinical appointment; (4) other; (5) don’t know. (5 responses)**

(1) 0 (2) 1 (3) 0 (4) 0 (5) 0

(1) 0 (2) 4 (3) 0 (4) 0 (5) 0

(1) 0 (2) 39 (3) 3 (4) 0 (5) 0

(1) 0 (2) 10 (3) 0 (4) 0 (5) 0

(1) 0 (2) 13 (3) 0 (4) 0 (5) 0

**What is the minimum time required to complete your DMP program? (5 responses)**

4 y

45 mo

4 y

4 y

4 y

**For your graduates, what is the average time required to complete the DMP program? (5 responses)**

6 y

45 mo

4 y

4 y

4 y

**How many students are currently enrolled in your DMP program? (5 responses)**

0

4

5

6

10

**How many students typically matriculate each year into your DMP program? (5 responses)**

0

2

1

3

3

**What is the maximum number of students who could ultimately matriculate each year into your DMP program? (5 responses)**

3

3

1

6

4

**What is the total tuition burden FOR THE ENTIRE DMP PROGRAM for an in-state student? (5 responses)**

$70,000

$40,000

~$150,000

$115,744

$84,000

**What is the total tuition burden FOR THE ENTIRE DMP PROGRAM for an out-of-state student? (5 responses)**

$125,000

$65,000

~$150,000

$209,680

$144,000

**Do you provide funding (scholarships, clinical employment, etc.) for your students during their clinical training? (5 responses)**

Yes

For most students

Yes

Yes

No

**If yes, please specify the mechanism and amount: (4 responses)**

Scholarship (proposed $10k for resident and $50k for nonresident total for years 3 and 4)

Graduate assistantships (tuition + annual stipend of $17k)

Third and fourth year stipends of $10,000 per student/per year

44,000 tuition remission divided among all students.

**Do you have a specific mechanism for a prior MS or PhD graduate from your institution to complete the DMP? (5 responses)**

Yes

Yes

No

No

Yes

**If yes, please describe: (3 responses)**

Students completing all didactic and research components of the program may apply 2 y of satisfactorily completed residency training from any CAMPEP accredited program toward the clinical training component of the DMP but must officially register for the coursework.

Fast-tracked to clinical rotation (after 2 semesters of didactic coursework).

The students must apply for admission into the DMP program. Then they must complete the didactic coursework in the DMP curriculum that they have not yet taken before entering the full clinical years.

**Would you allow a graduate student enrolled in your DMP program to enter the MedPhys Match? (5 responses)**

Yes

Yes

Yes

Yes

No

**If not, please explain why: (1 response)**

We do not offer a degree in the middle of the program that would satisfy the requirements of the MedPhys Match. After they graduate, they no longer need the Match process.

TA = teachers’ assistant; NA = not applicable.

**Table 1.** Selected data from the five accredited programs provided within the 2020 WGPDMP survey

|  | **1** | **2** | **3** | **4** | **5** | **Ave** |
| --- | --- | --- | --- | --- | --- | --- |
| Total credit hours required for the DMP | 90 | 80 | 92 | 118 | 98 | 95.6 |
| Credit hours representing the 2 y of clinical training | 30 | 40 | 36 | 44 | 52 | 40.4 |
| Required research credit hours | 12 | 4 | 6 | 17 | 1 | 8.0 |
| Credit hours of didactic coursework | 48 | 40 | 50 | 57 | 45 | 48.0 |
| Credit hours required for MS degree | 35 | 40 | 44 | 58 | NA | 44.3 |
| Credit hours required for PhD degree | 90 | NA | NA | NA | 72 | 81.0 |
| Professional skills training beyond that provided in MS/PhD | Yes | No | No | Yes | Yes |  |
| Minimum time to complete the DMP (y) | 4 | 3.75 | 4 | 4 | 4 | 4.0 |
| Average time to complete the DMP (y) | 6 | 3.75 | 4 | 4 | 4 | 4.4 |
| Average number of students matriculating per year | 0 | 2 | 1 | 3 | 3 | 1.8 |
| Maximum number of students that could matriculate per year | 3 | 3 | 1 | 6 | 4 | 3.4 |
| Total tuition for entire program for in-state student | $70k | $40k | $150k | $116k | $84k | $92k |
| Total tuition for entire program for out-of-state student | $125k | $65k | $150k | $210k | $144k | $139 |
| Total graduates as of 9/2020 | 1 | 4 | 42 | 10 | 13 |  |

NA = not applicable.
